# Pectin methylesterase gene *AtPMEPCRA* contributes to physiological adaptation to simulated and spaceflight microgravity in Arabidopsis

**DOI:** 10.1016/j.isci.2022.104331

**Published:** 2022-04-29

**Authors:** Peipei Xu, Haiying Chen, Jinbo Hu, Xiaocheng Pang, Jing Jin, Weiming Cai

**Affiliations:** 1 Laboratory of Photosynthesis and Environment, CAS Center for Excellence in Molecular Plant Sciences, Shanghai Institute of Plant Physiology and Ecology, Chinese Academy of Sciences, Shanghai 200032, China; 2 University of Chinese Academy of Sciences, Beijing 100039, China

**Keywords:** Biological sciences, Plant biology, Plant physiology, Space sciences

## Abstract

Pectin is biosynthesized in a highly methylated form and is partially de-methylated by pectin methylesterase (PME) activity. Plant PMEs play a critical role in cell wall remodeling in many physiological processes. Here, we studied Arabidopsis seedlings, which had been exposed to simulated or actual microgravity. Simulated microgravity inhibited total PME activity in Arabidopsis seedlings. We identified that *AtPMEPCRA* expression played a major role in the microgravity-induced inhibition of PME activity. *atpmepcra* mutants did not exhibit the enlarged leaf area of Arabidopsis seedlings observed under spaceflight microgravity. The downregulation of *AtPMEPCRA* expression in response to microgravity was due, in part, to changes in methylation patterns. The sexual offspring of the plants grown during spaceflight retained the methylation changes at *AtPMEPCRA* locus for one generation and thus contribute to the physiological adaptation to microgravity among F_1_ offspring seed generation. We conclude that *AtPMEPCRA* contributes to the spaceflight-induced transgenerational responses in Arabidopsis.

## Highlights

• Simulated microgravity inhibited total PME activity in Arabidopsis seedlings
• *AtPMEPCRA* played a major role in the microgravity-induced inhibition of PME activity
• *atpmepcra* mutants did not exhibit the enlarged seedlings leaf area observed under microgravity
• *AtPMEPCRA* regulates the spaceflight-induced transgenerational responses in Arabidopsis

## Graphical abstract

**Figure:**
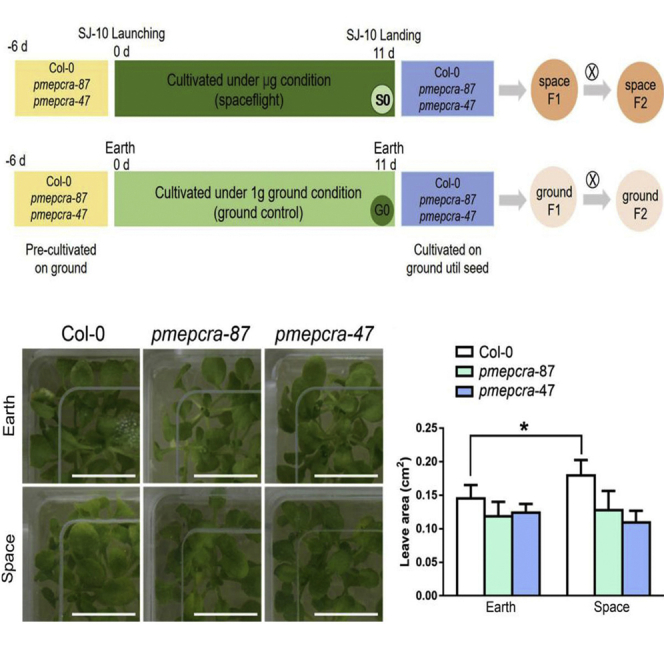

## Introduction

Plants are subjected to the constant effect of gravity on Earth, and plant evolution has also been affected by gravity (Monje et al., 2003). The gravity on Earth is approximately 9.8 m/s^2^, defined as 1 g. Generally, microgravity (1 μg) refers to an effective gravity level close to zero-g, resulting in weightlessness. In recent years, researchers have used machines known as Random Positioning Machines (RPMs), such as the three-dimensional clinostat, to manipulate gravity or simulate microgravity (Wuest et al., 2015). In these, rotation can constantly change the direction of gravity to maximally counteract the unilateral effect of gravity on Earth, although gravity does not actually decrease (Hoson, 2014). Simulated microgravity has little effect on the seed germination of many plant species, but it can influence morphogenetic and growth processes that depend on the direction of embryo orientation (Hoson et al., 2001, 2002).

Arabidopsis cell walls are mainly composed of pectin, cellulose, and hemicellulose (Zablackis et al., 1995). Pectin is a group of complex polysaccharides composed of xylogalacturonan, rhamnogalacturonan I (RGI), rhamnogalacturonan II (RGII), and homogalacturonan acid (HG) (Zablackis et al., 1995). Pectin is synthesized in the Golgi apparatus and secreted into the cell wall in a highly methyl-esterified form (Harholt et al., 2010; Kim et al., 2015). Pectin methylesterase (PME) is a ubiquitous cell-wall-related plant enzyme that is the first enzyme to act on pectin, promoting the de-esterification, modification of pectin in the plant cell wall (Micheli, 2001). The major role of PME is to catalyze the specific de-methylesterification of HG within the plant cell walls. The de-methylesterified HG can either form Ca^2+^ bonds in a so-called “egg-box” structure to form a gel or become a target for pectin-degrading enzymes, either route affecting cell wall structure and integrity. Therefore, PMEs play an essential role in cell wall pectin remodeling (Pelloux et al., 2007).

So far, very limited evidence has been reported to suggest that pectin can play a role in supporting tissue formation (Willats et al., 2001). Analysis of transcriptionally co-expressed genes indicates that there is an association between expression levels of pectin-related genes and the genes involved in secondary wall biosynthesis (Brown et al., 2005; Persson et al., 2005). Transcriptomic studies have shown that the expression of pectin-associated genes is altered during gravitropism (Hoson et al., 2002; Jin et al., 2015). Changes in cell wall composition in response to microgravity simulation has also been reported (Hoson et al., 2004; Yuda et al., 2000). The effect of microgravity on plant cell walls has been characterized by a decrease in cellulose and lignin contents (Perbal, 2003). These changes may increase the malleability of the cell wall to accommodate the response of plant tissues to microgravity (Hoson et al., 2002), data suggesting that pectin may be involved in the mechanical support of cell walls under microgravity.

PME plays an important role in pectin remodeling and cell wall decomposition in many physiological processes, such as plant-microbe interactions and stress responses. When challenged by microbial pathogens, the plant will locally trigger the strong induction of pathogen-induced PME activity (Lionetti et al., 2015). It is worth mentioning that the de-methylation and esterification of pectin by PME produces methanol, which is a plant alarm signal operating during the process of pathogen challenge/resistance (Dorokhov et al., 2012). However, the mechanisms by which transcriptional and post-transcriptional regulation of PME activity take place are still unknown.

PME activity is regulated posttranslationally by PME inhibitors (PMEIs) in plants (Lionetti et al., 2015; Senechal et al., 2015). In the Arabidopsis genome, there are 67 putative members of the *PME* gene family and 69 putative members of the *PMEI* gene family (Jolie et al., 2010). *PME*/*PMEI* genes regulate various processes in plant growth and development (Micheli, 2001). For instance, PME activity and secretion play important roles in cell elongation and pollen tube growth (Bosch et al., 2005). PME is also necessary for organ growth, with normal cell elongation in the Arabidopsis hypocotyl requiring at least about 60% pectin methylesterification (Derbyshire et al., 2007). Changes in PME activity may be associated with the brassinosteroid (BR) signaling pathway, which, among other processes, is involved in maintaining cell wall integrity during cell expansion (Wolf et al., 2012, 2014). The *pme46* mutant reduces the binding of aluminum to the cell wall, thereby reducing aluminum-induced root growth inhibition (Geng et al., 2017). The pectin methylesterification status also affects disease resistance in plants (Lionetti et al., 2017). An increase in PME activity was concomitant with a decrease in pectin methylesterification in response to pathogen challenge, with bacterial pathogen-induced PME activity being dependent on jasmonic acid (JA) signaling (Bethke et al., 2014). Genes *AtPMEI10*, *AtPMEI11*, and *AtPMEI12* were all involved in the maintenance of cell wall integrity in plant immunity (Lionetti et al., 2017). It was reported that short-term exposure to simulated microgravity reduced pectin content without any impact on cell wall thickness, but unfortunately the PME activity was not measured in this study (Yuda et al., 2000). PME35 regulates the pectin methylesterification status in the primary cell wall and also regulates cell wall stiffening in the Arabidopsis peduncle (Louvet et al., 2006).

PMEs are classified into two subfamilies: type I/group 2 and type II/group 1 (Jolie et al., 2010). Type I/group 2 PME isoforms contain an N-terminal PRO region, whereas type II/group 1 isoforms lack it (Micheli, 2001; Tian et al., 2006). In the current study, we used three-dimensional (3D) clinostat-induced simulated microgravity and microgravity associated with spaceflight in the SJ-10 recoverable satellite program and identified an Arabidopsis *PECTIN METHYLESTERASE* gene*, AtPMEPCRA,* which appeared to play an important role in plant adaptation to the spaceflight environment. Arabidopsis AtPMEPCRA (At1g11580), which belongs to the type I PME subfamily, has both the PME catalytic domain and the PMEI domain. Previous work had shown that expression of the *AtPMEPCRA* gene was markedly induced by the herbicide isoxaben in cultured Arabidopsis cells (Manfield et al., 2004) and in distal leaf tissues after inoculation with the fungal pathogen *Alternaria brassicicola* (Schenk et al., 2003). Microarray data showed that expression of *AtPMEPCRA* was upregulated in response to inoculation with *Pseudomonas syringae* pv. *Maculicola* (Wang et al., 2008), with subsequent research confirming this result (Bethke et al., 2014). However, the function of the *AtPMEPCRA* gene in plant growth and development, hormone signaling, and response to simulated microgravity stress are largely unknown.

Here, we found that simulated microgravity significantly inhibited seedling PME activity and, through checking the response to microgravity of members of the PME type I subfamily, determined that *AtPMEPCRA* played a central role in this process. Focusing on the function of the *AtPMEPCRA* gene in relation to Arabidopsis adaptation to the spaceflight microgravity environment, we showed that changes in the methylation status of *AtPMEPCRA* occurred not only in the generation grown under spaceflight conditions but also in its seed offspring grown on Earth. The retention of the *AtPMEPCRA* methylation status could result in alteration of the gene expression pattern and thus contribute to the physiological adaptation to microgravity among the F_1_ offspring seed generation. In summary, we demonstrated that the *AtPMEPCRA*-mediated PME activity is involved in spaceflight-induced transgenerational adaptive responses in Arabidopsis.

## Results

### Simulated microgravity treatment of Arabidopsis seedlings inhibits total PME activity

**Figure 1:**
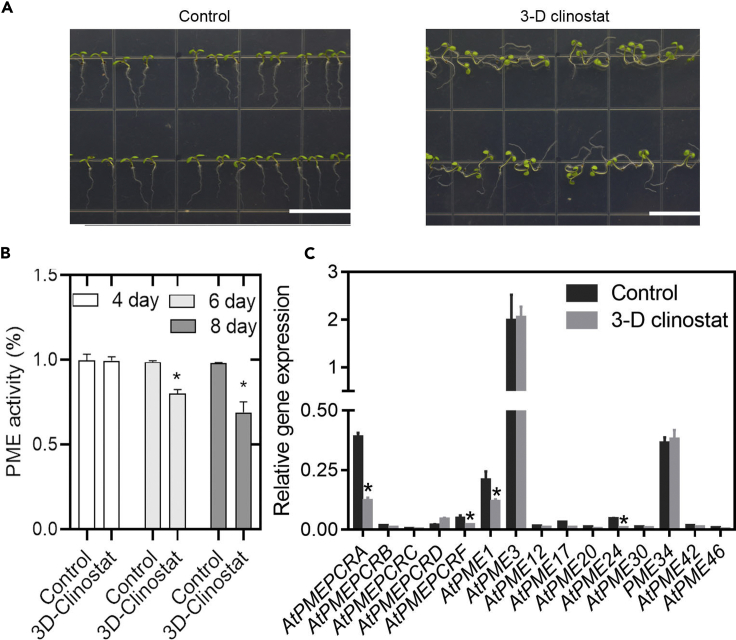
Seedling pectin methylesterase (PME) activity and *AtPMEPCRA* expression were inhibited under simulated microgravity treatment (A) Wild-type Col-0 plants were exposed to simulated microgravity in a 3D clinostat. Root growth after 8-day simulated microgravity treatment was nondirectional, but root length was not significantly different from that of gravity-treated control plants. (B) Seedlings were harvested after a few days of simulated microgravity treatment. The total protein was extracted in the control and simulated microgravity seedlings at each time point, and the activity of PME was determined. Bars indicate SE. ∗ indicates significant differences (*p*＜0.05), using Student’s t test. (C) qRT-PCR analysis of gene expression pattern of some PME subfamily genes in Arabidopsis after exposure to simulated microgravity for 8 days. Bars indicates SE from three independent experiments, and three technical repetitions were performed for each replicate. ∗ indicates significant differences (*p*＜0.05), using Student’s t test.

A three-dimensional (3D) clinostat had previously been constructed to reduce the directionality of gravitation pull (Pardo et al., 2005). The rate of seed germination of various plant species had been shown to be unaffected by the clinostat (Kiss et al., 2019), but, to determine the effects, if any, of simulated microgravity on the PME activity of Arabidopsis seedlings, we tested the PME activity of the seedlings after 8 days of exposure to simulated microgravity processing in a 3D clinostat. The root growth of Arabidopsis Col-0 wild type after 8 days of simulated microgravity showed no directionality, although there was no significant difference in root length compared with the control seedlings (Figures 1A and S1). From 4 days after treatment started, total PME activity was measured every 48 h. Significant inhibition of total PME activity was observed in the microgravity-treated seedlings from 6 days onward after the start of the simulated microgravity treatment (Figure 1B).

### Inhibition of PME activity by simulated microgravity is partly abscisic acid dependent

**Figure 2:**
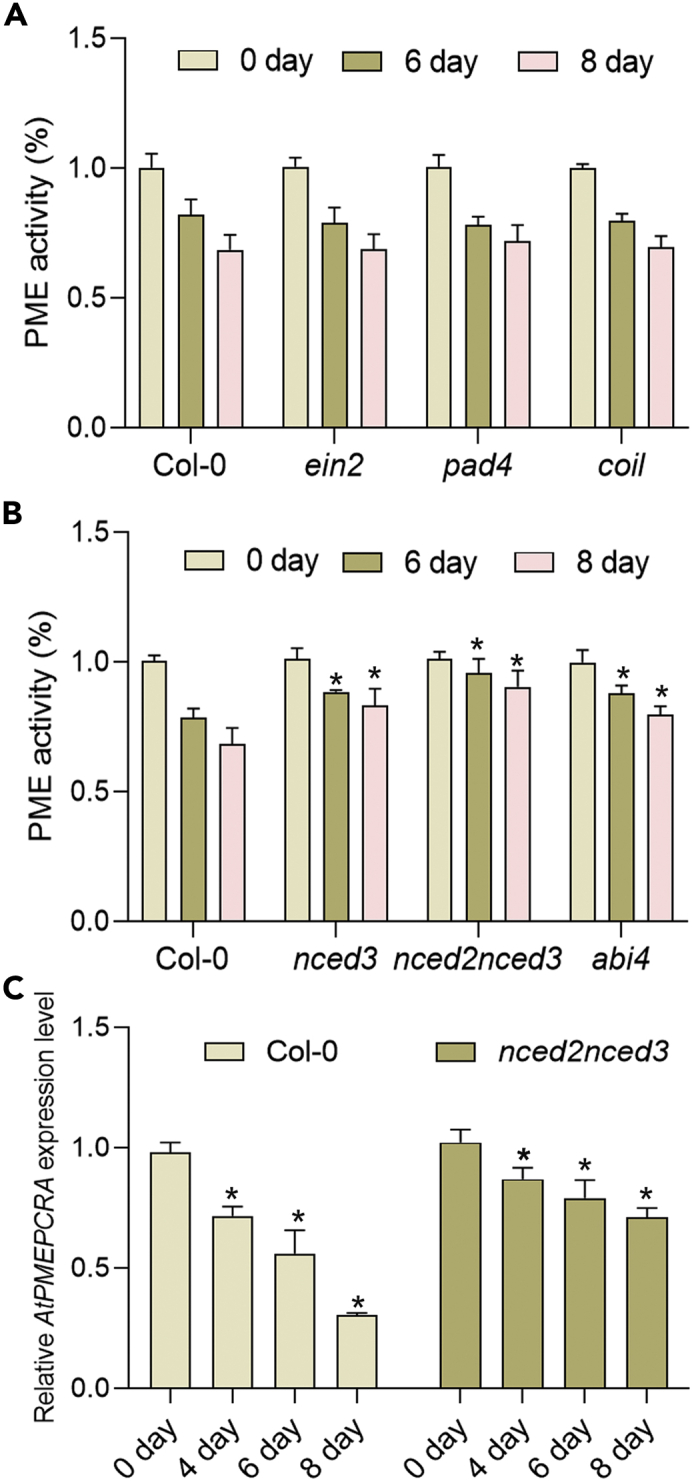
Effect of simulated microgravity on PME activity in mutants blocked with respect to different plant hormone pathways (A) Plants of wild type and mutants defective in jasmonic acid (JA) perception (*coi1*), ethylene (ET) signaling (*ein2*), salicylic acid (SA) signaling (*pad4*), and (B) abscisic acid (ABA) biosynthesis (*nced3, nced2nced3*) and ABA signaling (*abi4*) were exposed to simulated microgravity. Seedlings were harvested from 0 days to 8 days after treatment began and total PME activity was assayed. (C) Inhibition of *AtPMEPCRA* expression level by simulated microgravity was significantly attenuated in the ABA biosynthesis *nced2nced3* double mutants. Bar indicates SE from three independent biological replicates. ∗ indicates significant differences (*p*＜0.05), using Student’s t test.

PME activity is related to pectin integrity and plant growth, so the decrease in PME activity in response to simulated microgravity can be considered to be part of the adaptive response in plants. Plant hormones play a key role in adaptive responses, as central mediators to coordinate response to different environmental signals. To identify which (if any) hormones were involved in the microgravity response, we measured the PME activity in microgravity-exposed seedlings of wild-type and mutant plants with defects in the main part of different hormone signaling networks, namely *coi1* (blocked in jasmonic acid [JA] signaling), *sid2* (blocked in salicylic acid [SA] signaling), *ein2* (blocked in ethylene [ET] signaling), *abi4* (required for abscisic acid [ABA] signaling), and *nced3*, *nced2nced3* (required for ABA biosynthesis). The inhibitory effect of simulated microgravity on PME activity was significantly alleviated in ABA hormone pathway mutants compared with the control (Figure 2A), but no significant change in the inhibition of PME activity was observed in the other mutants compared with the wild type. In summary, the inhibitory effect of microgravity on PME activity in *ein2*, *coi1*, and *sid2* mutant plants was similar to that of the wild type, except for *abi4* and *nced2nced3*, which are involved in signaling and biosynthesis aspects, respectively, of the ABA hormone pathway (Figure 2B). In the control samples, none of the mutants exhibited total PME activities significantly different from that of the wild type. In addition, we further performed the horizontal rotation control provided by a single axis of the clinostat. The results showed that after 3–8 days of horizontal rotation treatment of the Col-0 samples, similar to the ground control, the relative PME activity and expression patterns of *AtPMEPCRA* gene in Arabidopsis seedlings could not be significantly changed Figure S2). So, we got the conclusion that the simulated microgravity stimulation induced by 3D clinostat resulted in the inhibition of PME activity. These results indicated that it is the phytohormone ABA, rather than ET, SA, or JA, that was involved in the simulated microgravity-induced inhibition of the PME activity.

### Expression of the *AtPMEPCRA* gene is inhibited by simulated microgravity

In order to identify which member(s) of the PME gene family contributed to the inhibited PME activity in response to simulated microgravity, we examined the transcriptional expression levels of numerous PME subfamily members, including *AtPMEPCRA, AtPMEPCRB, AtPMEPCRC, AtPMEPCRD, AtPMEPCRF, AtPME1, AtPME3, AtPME12, AtPMEPCRA, AtPME20, AtPME24, AtPME30, AtPME34, AtPME42,* and *AtPME46* in Col-0 backgrounds in the presence or absence of simulated microgravity treatment, using the real-time qPCR (RT-qPCR) method. Of these genes, *AtPMEPCRA, AtPMEPCRF, AtPME1, AtPMEPCRA,* and *AtPME24* exhibited lower expression after microgravity treatment, compared with the untreated control, especially *AtPMEPCRA*, where the expression level was one-third that of the control; the other genes tested displayed no significant difference in the expression level compared with the corresponding untreated control (Figure 1C). Given that the most severely inhibitory effect of simulated microgravity was on the expression of the *AtPMEPCRA* gene, we then checked its expression level in response to simulated microgravity treatment in the ABA pathway mutants. The effect of simulated microgravity on *AtPMEPCRA* expression was obviously alleviated in these mutants’ background due to the fact that the reduction of *AtPMEPCRA* expression is not abolished in the ABA mutant background (Figure 2C). Therefore, we reached the conclusion that simulated microgravity-inhibited *AtPMEPCRA* expression was a partly ABA-dependent effect.

### Molecular and biochemical characterization of *AtPMEPCRA* gene in Arabidopsis

**Figure 3:**
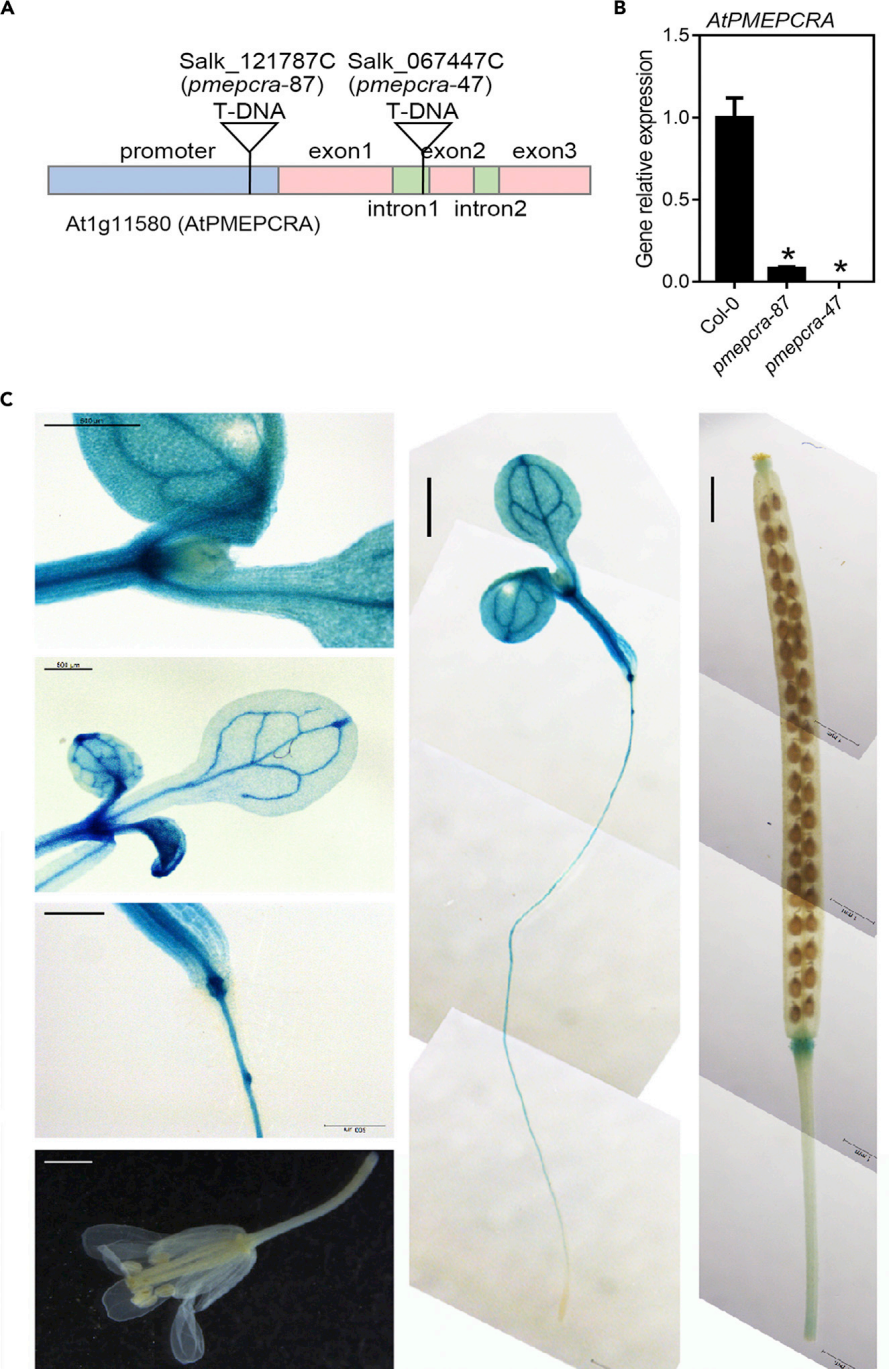
Identification of *atpmepcra* transfer DNA insertion mutants and molecular analysis of the *AtPMEPCRA* gene (A) Organization of the *AtPMEPCRA* gene. The pink boxes show the positions and sizes of the exons. The green boxes show the positions and sizes of the introns. Each triangle indicates a site of transfer DNA insertion. (B) Identification of relative *AtPMEPCRA* gene expression level in Col-0 and mutants. Bar indicates SE. ∗ indicates significant differences (*p*＜0.05), using Student’s t test. (C) proGUS analysis of *AtPMEPCRA* expression patterns in various organs: leaf vein, cotyledon, peduncle, root tip, root, and silique.

Two transfer DNA insertion lines of *AtPMEPCRA* were ordered from The Arabidopsis Information Resource (TAIR). The transfer DNA insert position of *pmepcra-87* (Salk_121787) was in the promoter region, whereas that of the *pmepcra-47* line (Salk_067447) was in the first intron (Figure 3A). *AtPMEPCRA* gene expression of the *pmepcra-87* line was about 95% knocked down, whereas that of *pmepcra-47* was knocked out (Figure 3B). Each of the two insertion lines showed significantly lower PME activity than the Col-0 wild type (Figure S3A). In order to determine the spatial expression pattern of *AtPMEPCRA*, approximately 2.5 kb of the promoter region of *AtPMEPCRA* was fused to the β-glucuronidase (GUS) reporter gene. The GUS signal was observed in the cotyledon, leaf vein, hypocotyl, lateral root primordium, and primary root (Figure 3C). However, GUS was not detected in the root tip, flower, nor the silique (Figure 3C). These expression patterns were consistent with the RT-qPCR results (Figure S3B). The pPMEPCRA:GUS expression pattern did not change after simulated microgravity treatment, but the GUS level was significantly suppressed (Figure S4).

We then decided to obtain a recombinant AtPMEPCRA protein, in order to verify whether AtPMEPCRA was a functional enzyme and to achieve a greater in-depth understanding of its possible role in the modification of cell wall pectin in the process of simulated microgravity processing. Using the cDNA obtained from the mRNA extracted from the leaves of *Arabidopsis thaliana*, the AtPMEPCRA coding sequence was amplified by PCR, then cloned into the pET30a expression vector and expressed in *Escherichia coli*. The method of Ni-NTA affinity chromatography agarose beads was used to purify AtPMEPCRA protein and then PME activity was detected.

**Figure 4:**
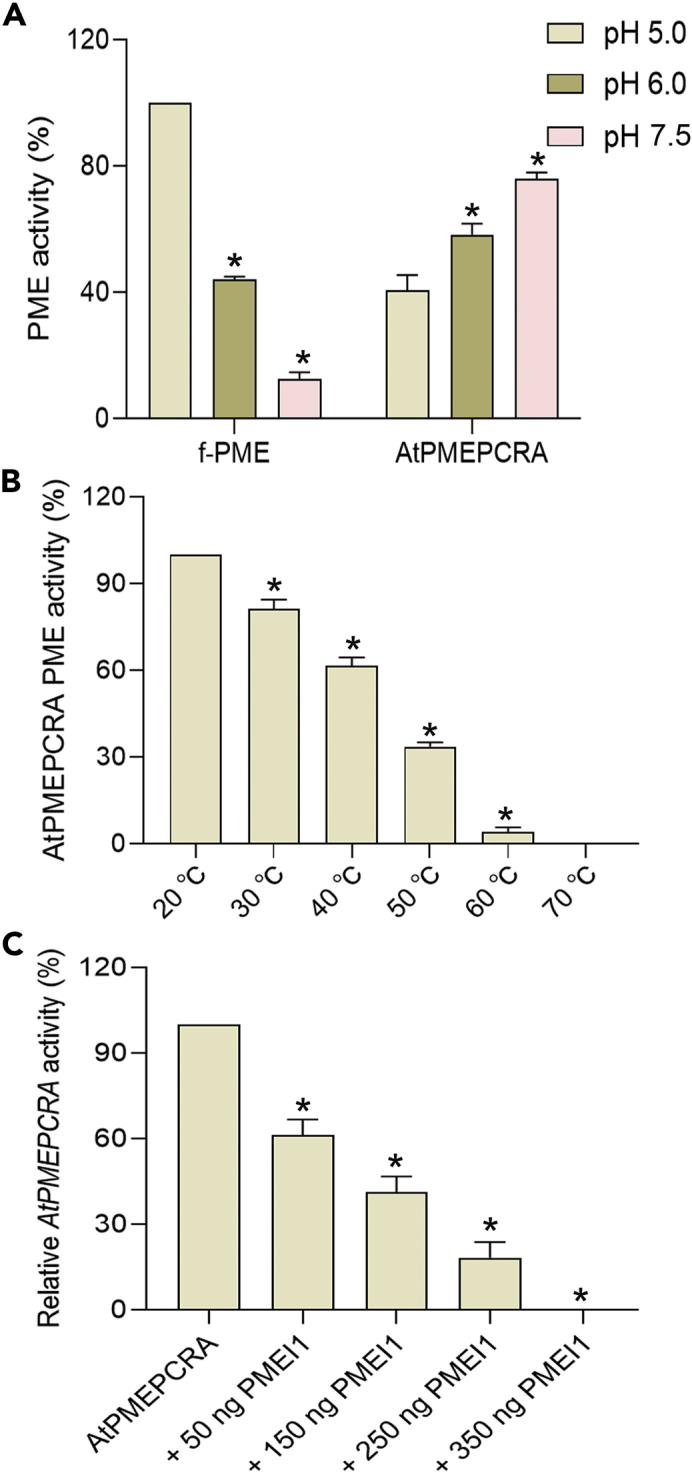
Sensitivity of *AtPMEPCRA* pectin methylesterase (PME) activity to pH, temperature, and pectin methylesterase inhibitor (AtPMEI1) (A) The response to pH of purified recombinant AtPMEPCRA and *Botrytis cinerea* PME (f-PME) was compared. Bar indicates SE. ∗ indicates significant differences (*p*＜0.05), using Student’s t test. (B) Thermal stability of purified recombinant AtPMEPCRA. The activity at 30°C was set at 100%, and the activities incubated at different temperatures were expressed relative to that at 30°C. Bar indicates SE. ∗ indicates significant differences (*p*＜0.05), using Student’s t test. (C) Inhibition of purified recombinant AtPMEPCRA by AtPMEI1. Values are expressed as means ± SE from three independent biological replicates. ∗ indicates significant differences (*p*＜0.05), using Student’s t test.

Studies on the recombinant AtPMEPCRA protein predicted that the protein consists of an N-terminal PRO region and a C-terminal catalytic PME domain. We obtained the recombinant AtPMEPCRA protein to identify its optimum working conditions at different pH values and temperatures. We used *Botrytis cinerea* PME (*f*-PME) for comparison when testing the activity of purified AtPMEPCRA at different pH levels. From acidic pH 5.5 to pH 7.5, AtPMEPCRA activity increased with increasing medium pH; in contrast, the *f*-PME activity of *B. cinerea* was highest at the lowest pH, decreasing under moderately alkaline conditions, and was lowest at pH 7.5 (Figure 4A). The thermostability of the recombinant AtPMEPCRA was also evaluated by testing the enzymatic activity after preincubation at elevated temperatures. AtPMEPCRA activity remained stable up to 40°C. However, as the temperature increased from 50°C to 60°C, the activity decreased significantly, with AtPMEPCRA activity being completely inactivated at 70°C (Figure 4B). AtPMEI1 inhibited AtPMEPCRA activity with maximum inhibition at the high concentration (Figure 4C).

### The enlarged leaf area of Arabidopsis seedlings grown under SJ-10 spaceflight involves AtPMEPCRA

**Figure 5:**
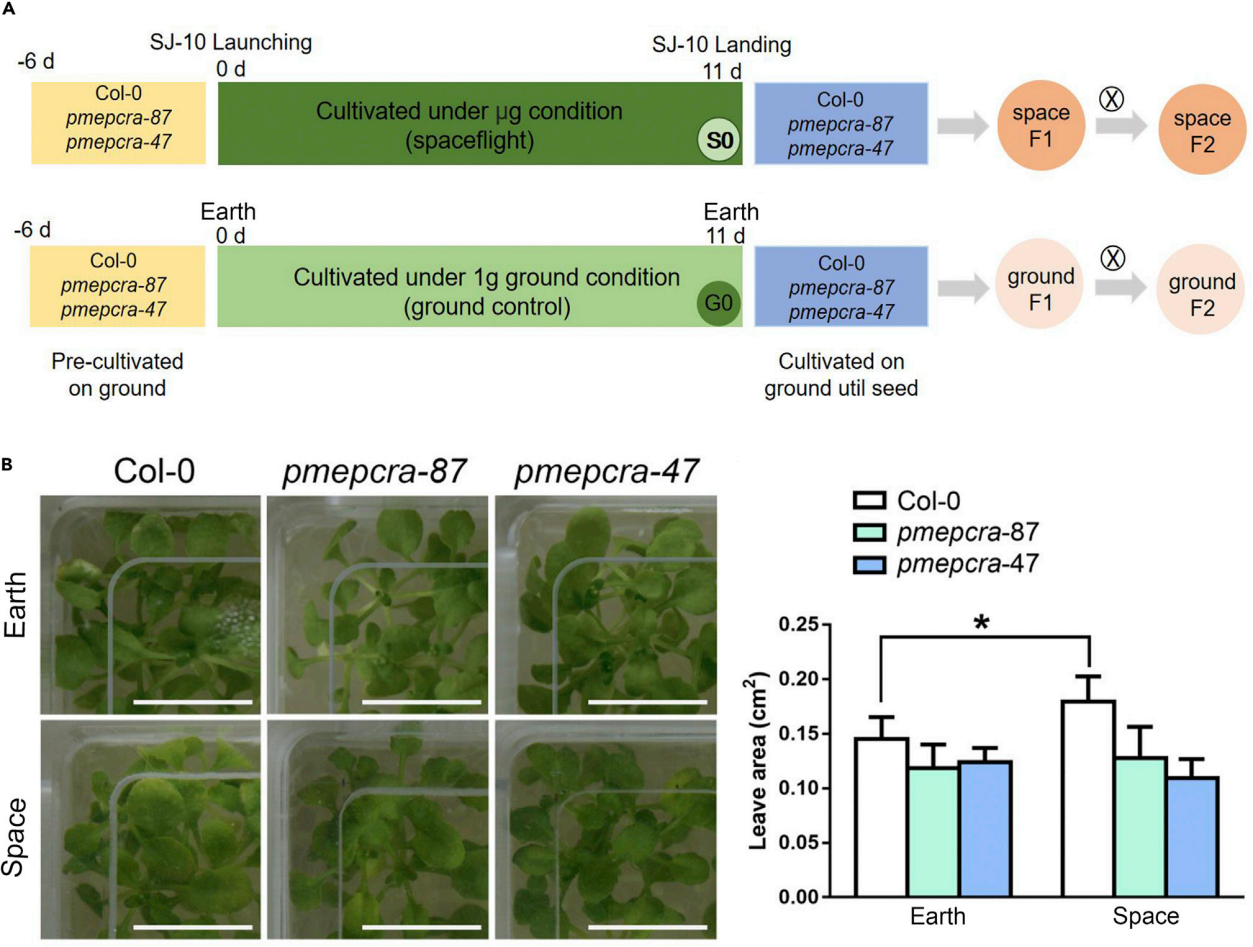
Comparison of mean leaf area of seedlings of Arabidopsis Col-0 wild type and *atpmepcra* transfer DNA insertion mutants grown under microgravity or gravity (A) Seedlings were grown on Earth (exposed to gravity) or in space on the SJ-10 satellite (exposed to microgravity) for 11 days. Bar: 1 cm. (B) The data shown are the mean values ±SD obtained for at least eight leaves from the plants presented in (A). The leaf area was measured using ImageJ. Bar indicates means ± SE from three independent biological replicates. ∗ indicates significant differences between the same genotype exposed to gravity of microgravity (*p*＜0.05), using Student’s t test.

In view of the possible important role of the *AtPMEPCRA* gene in response to simulated microgravity, we questioned whether it could also be involved in plant adaptation to the true microgravity environment of spaceflight. We tested this hypothesis, using the Chinese recoverable scientific satellite SJ-10. We obtained seedlings of the Col-0 wild type and the two T-insertion lines *pmepcra*-87 and *pmepcra*-47 after growing for 11 days under spaceflight microgravity in the cultivation unit 2 of the SJ-10 satellite (Xu et al., 2021; Xu et al., 2018). The seedlings were photographed immediately after removal from the satellite to measure the area of individual leaves. At least eight true leaves of each Arabidopsis seedling grown under microgravity and control Earth (gravity) conditions were used for measurement at the same leaf position. The results showed that the mean area of leaves of the Col-0 seedlings grown under true microgravity conditions were significantly larger than those of the Earth-grown control plants of the same genotype (Figures 5A and 5B). However, the leaves of the *atpmepcra-87* and *atpmepcra-47* mutants showed no significant difference in area between seedlings grown in space and those grown on Earth (Figure 5B). The spaceflight experiments suggest that the leaves of Arabidopsis seedlings grown under microgravity generate larger leaves than those on seedlings grown on Earth and that this process requires the functionality of the *AtPMEPCRA* gene, indicating a positive role for the *AtPMEPCRA* gene in regulating leaf growth and morphogenesis in achieving adaptation to microgravity conditions.

### Identification of spaceflight microgravity-induced heritable DNA methylation pattern changes at *AtPMEPCRA* locus

The spaceflight experiments showed that Arabidopsis leaves grew larger under microgravity. This poses a further question as to whether this effect of microgravity could persist in the next generation (as an epigenetic effect), as we know that epigenetic characteristics play a key role in controlling gene expression, with a subsequent adaptive response to their environment. The seedlings grown in the satellite under microgravity for 11 days were still alive and vigorous when they returned with the satellite (Xu et al., 2021; Xu et al., 2018). We collected their seeds as the F_1_ generation for further analysis. We had previously mapped changes in the methylation pattern of the Arabidopsis genome in response to microgravity conditions in the satellite SJ-10 at the level of single-base resolution (Xu et al., 2018), with Arabidopsis seedlings exposed to the spaceflight microgravity showing altered DNA methylation levels in the contexts of CHG, CHH, and CpG islands (Xu et al., 2018). It has been reported that cell wall rigidity is upregulated in stems and roots under hyper gravity (Martzivanou and Hampp, 2003), with plant cell wall rigidity also changing under a (simulated) microgravity environment (Hoson, 2014). We further tested for changes of the DNA methylation patterns of the PME family genes, especially the *AtPMEPCRA* gene, in response to the microgravity environment encountered during spaceflight.

**Figure 6:**
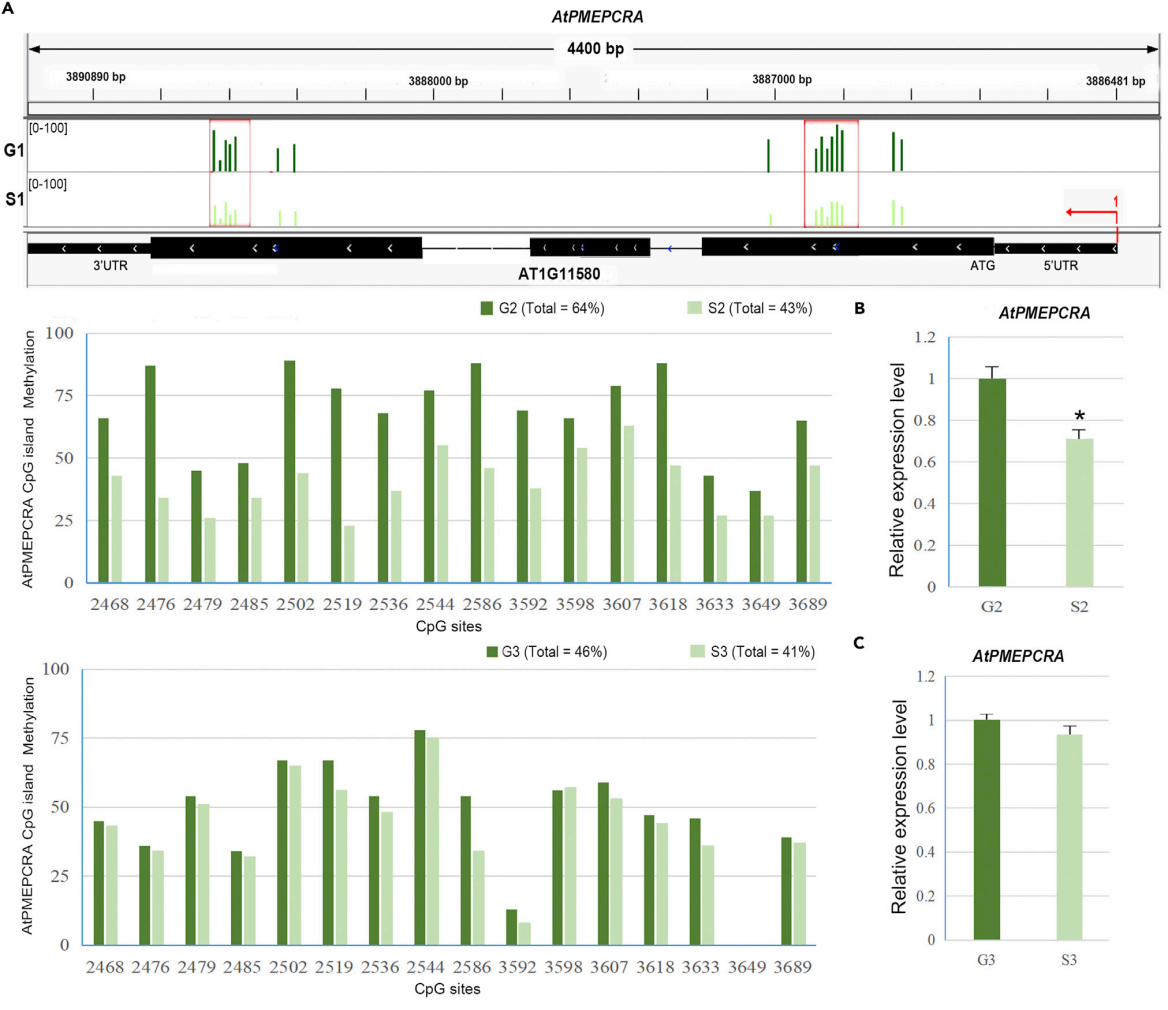
Analysis of spaceflight-induced heritable DNA methylation of *AtPMEPCRA* and analysis of the *AtPMEPCRA* expression levels in the offspring (A) Visualization of bisulfite sequencing PCR (BSP) analysis of *AtPMEPCRA* putative spaceflight-induced heritable differential methylated regions (DMRs). Visualization of methylation data was performed, using the Integrated Genome Viewer (IGV) (http://software.roadinstitute.org/software/igv). The inherited methylation level of CpG islands was verified using BSP analysis. (B and C) (B) Expression levels of *AtPMEPCRA* in the F_1_ and F_2_ (C) offspring of plants grown in space or on Earth. Parent plants were grown in space or on Earth for 11 days, and seed was collected from plants on Earth. Error bars are SE. ∗ indicates statistically significant difference using Student’s two-tailed t test (p < 0.05).

The *AtPMEPCRA* gene in seedlings that had been on the SJ-10 spaceflight showed altered DNA methylation patterns compared with the control seedlings that had been grown on Earth. Our previous results had shown that changes in genomic methylation levels resulting from the spaceflight environment can likely be passed on to future generations and hence affect plant population adaptability (Xu et al., 2021). Therefore, we speculated that changes in methylation levels of the *AtPMEPCRA* gene may be inherited, so we checked the methylation levels of the *AtPMEPCRA* gene in the F_1_ and F_2_ generations after spaceflight, and the results showed that the observed decrease in methylation levels of the *AtPMEPCRA* exon was indeed partially conserved in the F_1_ generation but not in the F_2_ generation (Figure 6A). The low level of methylation observed in the *AtPMEPCRA* exon could lead to reductions in the expression level of the *AtPMEPCRA* gene in the F_1_ generation from plants grown in space compared with the F_1_ generation from plants grown on Earth (Figure 6B). The methylation level of the *AtPMEPCRA* gene in the F_2_ generation was reduced to a large extent compared with the F_1_ generation of the plants grown in space, and the *AtPMEPCRA* gene expression level of the F_2_ generation was almost indistinguishable from that of the population that was grown on Earth, unlike the F_1_ generation (Figure 6).

### The *AtPMEPCRA* gene is required for the altered phenotype of the F_1_ generation from the space-grown parents

**Figure 7:**
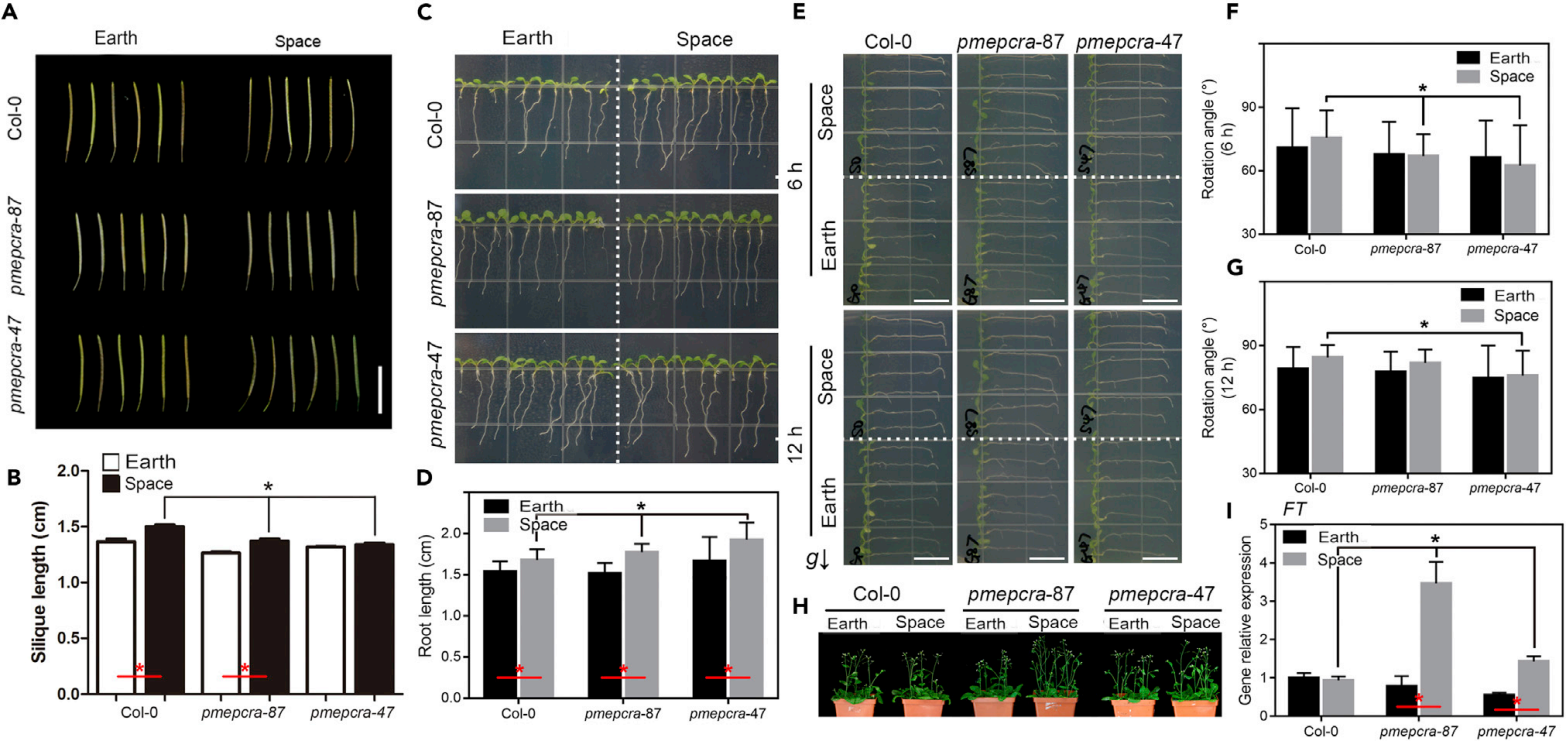
Comparison of the silique length, root length, flowering time, and gravitropic orientation analysis in F_1_ offspring of plants of Col-0 and *atpmepcra* transfer DNA insertion mutants grown in space or on Earth (A and B) The silique length and (C and D) root length of the F_1_ offspring of plants grown for 11 days in space or on Earth. Lengths were measured using ImageJ software. The data shown in b. and d. are the mean ± SE values obtained for about 21 plants per sample. The red bar indicates significantly different. (E–G) Analysis of the response to 90° change in gravitropic orientation analysis of the 8-day-old F_1_ offspring seedlings of the plants grown in space or on Earth. (H) Flowering time analysis of the F_1_ offspring of plants grown for 11 days in space or on Earth. (I) RT-qPCR analysis of FT expression levels. The data shown are the mean ± SE (n > 10). ∗ indicates a statistically significant difference between the samples, using Student’s t test (p < 0.05). Bar indicates 1 cm.

The F_1_ generation from the seedlings that returned from space exhibited longer roots than did the corresponding generation from the parent seedlings that grew on Earth (Figures 7A and 7B). To study the response of the seedlings to gravity, the F_1_ generation seedlings were displaced by 90° and assessed for root rotation angle after reorientation for 6 and 12 h (Figures 7C–7G). The rotation angle of mutant *atpmepcra-47* F_1_ seedlings from microgravity-grown parent seedlings at both 6 and 12 h was significantly smaller than that of the corresponding Col-0 wild-type F_1_ seedlings, but there was no significant difference between Col-0 and *atpmepcra-47* F_1_ seedlings from gravity-grown parent seedlings at either time period.

Furthermore, *atpmepcra-47* F_1_ seedlings from microgravity-grown parent seedlings flowered significantly earlier than Col-0 F_1_ seedlings from microgravity-grown parent seedlings but not earlier than F_1_ seedlings from gravity-grown parental seedlings (Figure 7H). *FLOWERING LOCUS T* (*FT*) encodes a small mobile protein that plays an essential role in the regulation of flowering time (Wigge, 2011), and expression levels of *FT* were determined in Col-0 and *atpmepcra-47* seedlings by RT-qPCR. *FT* gene expression was upregulated in F_1_ seedlings from microgravity-grown *atpmepcra* parents that flowered early (Figure 7I). Our preliminary conclusion is that the transfer DNA insertion mutations in the *AtPMEPCRA* gene can at least partly recover the spaceflight microgravity effect on flowering. Under gravity-based growth conditions, the *atpmepcra* mutant plants phenotype was rather small (Figure 7). However, the mutation appears to be able to specifically affect microgravity response in terms of changes in leaf area, flowering time, and the root growth parameters. It appears that the *AtPMEPCRA* gene may be specifically involved in the adaptation of plants to the microgravity environment associated with spaceflight.

### *AtPMEPCRA* knockout gene mutation upregulates ABA pathway and affects plant adaptation to spaceflight

**Figure 8:**
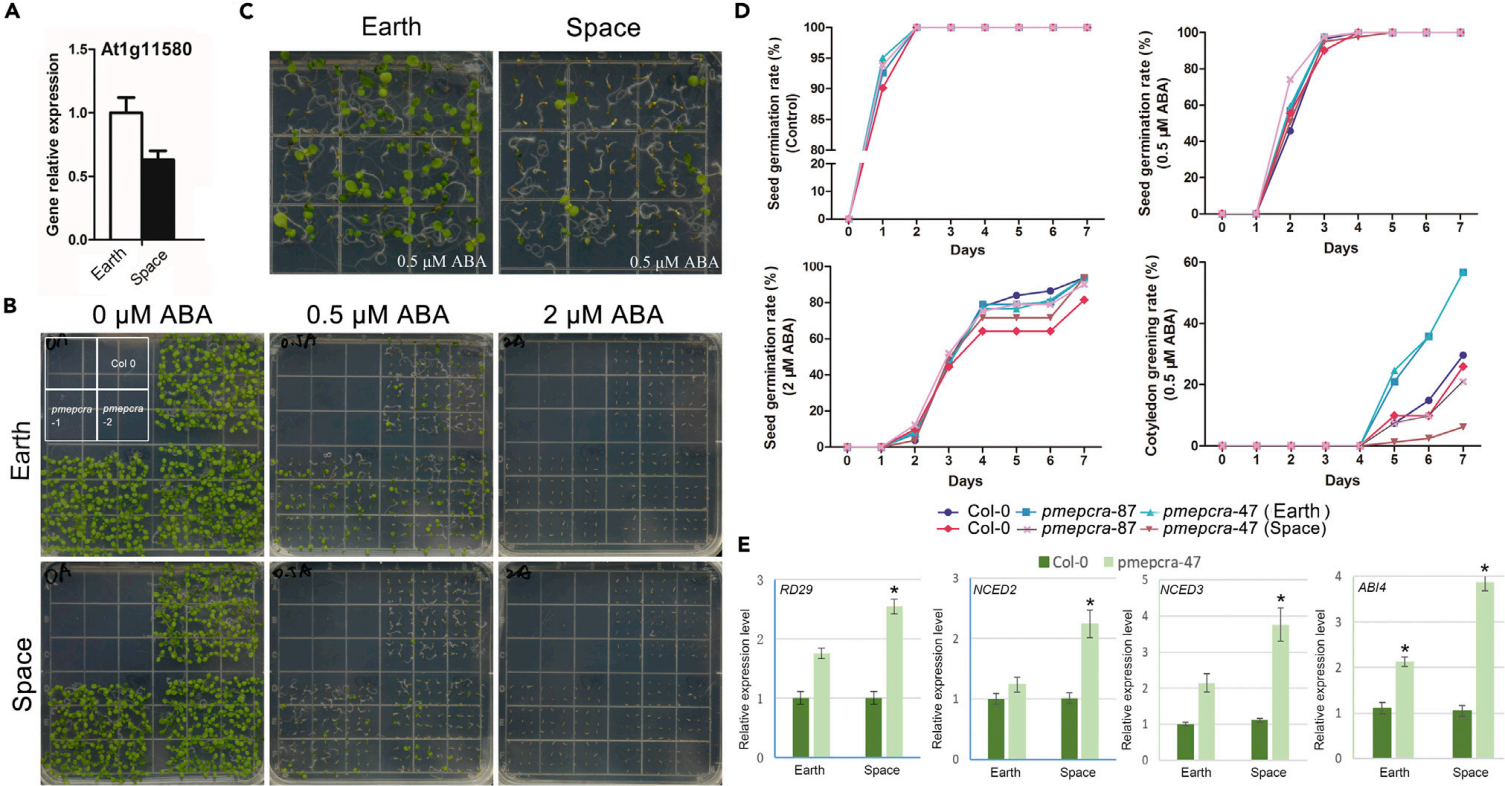
Analysis of gene expression levels and ABA response in the F_1_ offspring of plants of Col-0 and *atpmepcra* transfer DNA insertion mutants grown for 11 days in space or on Earth (A) Expression level of *AtPMEPCRA* in F_1_ generation of plants grown for 11 days in space or on Earth. B, c. Visualization of ABA response in the F_1_ generation. (B) and F_2_ generation. (C) of plants grown for 11 days in space or on Earth in the presence of various concentrations of ABA (n > 40). Bars indicate 1 cm. (D) Time course of seed germination of wild type and *atpmepcra* transfer DNA insertion mutants in the absence (CK) or the presence of various concentrations of ABA (n > 30 per sample). (E) Expression levels of ABA-associated genes in F_1_ generation of plants grown for 11 days in space or on Earth. Error bars indicate SE. ∗ indicates statistically significant difference using Student’s t test (p < 0.05).

We had previously shown, using mutants, that ABA is important in achieving full expression of microgravity-inhibited PME activity (Figure 2). Furthermore, treatment of seedlings with exogenous ABA significantly reduced the expression of *AtPMEPCRA* (Figure 8A). To identify the *AtPMEPCRA* gene function in the ABA signaling pathway in Arabidopsis, we grew F_1_ seeds of Col-0 and the *atpmepcra-47* transfer DNA insertion mutant from microgravity-grown parents on solid half-strength Murashige & Skoog (½ MS) medium with 0, 0.5, or 2 μM ABA added, for seven days. F_1_ seedlings of Col-0 and *atpmepcra-47* on 0 or 0.5 μM ABA medium displayed no significant difference between the two genotypes; however, in the presence of 2 μM ABA, F_1_ Col-0 seeds germinated much faster than did F_1_ *atpmepcra-47* seeds (Figures 8B–8D). Compared with Col-0, however, the F_1_ seedlings of the *atpmepcra-47* mutant showed a higher cotyledon greening rate in the presence of ABA stress, whereas the F_1_ seeds and seedlings from the microgravity-grown mutant plants showed a significantly lower germination rate and greening rate, respectively, than did the F_1_ seedlings from the gravity-grown mutant parents (Figure 8D).

Using RT-qPCR analysis, we compared expression patterns of ABA-associated genes in Col-0 and *atpmepcra-47* mutant. The genes studied included stress-induced genes *AtRD29* gene, expression of which is significantly induced by exogenous ABA treatments (Yamaguchi-Shinozaki and Shinozaki, 1993), and *AtNCED2* and *AtNCED3*, which are key genes in the ABA bio-synthetic pathway (Boursiac et al., 2013). The ABA signaling pathway gene *ABI4* is an ERF/AP2 transcription factor protein, and ABI4 involved in ABA-mediated glucose response and hexokinase-dependent sugar responses (Piskurewicz et al., 2008). We compared the expression patterns of these ABA-associated genes in F_1_ seedlings from microgravity- and gravity-grown wild type and mutant parent plants. The expression of *AtRD29* and *NCED2/3* did not differ in F_1_ seedlings between Col-0 and *pmepcra* mutant from gravity-grown plants (Figure 8E). All four genes had higher expression levels in F_1_ from microgravity-grown *pmepcra* mutant than that in the F_1_ from Col-0 plants (Figure 8E). As a result, among microgravity-grown F_1_ seedlings, the increase in the ABA pathway genes in the mutant is more significant. In summary, the *AtPMEPCRA* gene transfer DNA insertion mutant exhibited upregulation of expression of ABA biosynthesis genes, suggesting that feedback resulting from PME activity perturbation stimulated the ABA biosynthesis pathway.

## Discussion

The plant cell wall can protect cells from various stresses and provide structural support for plant growth and development (Harholt et al., 2010). In these different processes, maintaining cell wall integrity and function is a prerequisite for the cell wall to perform its specific functions. Existing evidence shows that there is a maintenance mechanism that can detect damage to the integrity of the cell wall and initiate a compensatory response to maintain function in plants (Lionetti et al., 2017). Pectin is one of the main components of cell walls in plants (Zablackis et al., 1995). Pectin is synthesized in the Golgi apparatus and secreted into the cell wall in a highly methylesterified form (Harholt et al., 2010; Kim et al., 2015). Genes encoding pectin methylesterase (PME) exist in a multi-gene family, encoding isoforms with different modes of action by removing methyl esters to promote the modification and subsequent decomposition of plant cell walls in many physiological processes (Micheli, 2001; Wormit and Usadel, 2018).

Using true (with the SJ-10 recoverable satellite) and simulated microgravity (induced by a 3D clinostat), we screened for and identified an Arabidopsis *PECTIN METHYLESTERASE* gene*, AtPMEPCRA,* which proved to play an essential role in plant adaptation to the spaceflight environment. Through quantifying the expression of multiple genes in the PME subfamily, we found that simulated microgravity significantly inhibited seedling PME activity, with *AtPMEPCRA* making a major contribution to this process. Tissue-specific expression analysis indicated *AtPMEPCRA* was ubiquitously expressed in most tissues. To explore the contribution of AtPMEPCRA to Arabidopsis total PME activity and the plant’s response to the spaceflight environment, AtPMEPCRA transfer DNA knockout and severe knockdown mutants were isolated. Growing under gravity, these mutants displayed no significant differences from the wild type in terms of vegetative growth. Interestingly, the F_1_ offspring of these *atpmepcra* mutants inhibited the enlarged leaf area and various physiological adaptive changes of Arabidopsis seedlings previously observed on wild-type seedlings grown on an SJ-10 spaceflight. It is important to mention that both transfer DNA mutants exhibited PME activity more than 60%, lower than that in the wild type, showing that *AtPMEPCRA* contributes to a major extent to the inhibition of total PME activity induced by simulated microgravity. No significant differences in growth phenotype were observed between the transfer DNA mutants and the wild type grown on Earth, showing that *AtPMEPCRA* functions specifically in response to spaceflight microgravity-grown plants. This study has therefore revealed a new function of *AtPMEPCRA* in regulating PME activity and its involvement in plant adaptation to growth under conditions of spaceflight-induced microgravity.

The molecular characterization of AtPMEPCRA was achieved through the heterologous expression of *AtPMEPCRA* in *E. coli*. Our *in vitro* biochemical studies showed that the recombinant protein expressed in *E. coli* exhibited PME activity, indicating that *AtPMEPCRA* is a functional Arabidopsis isoform. By assessing the effect of methyl ester distribution on cell wall degradation, we then evaluated the mode of action of AtPMEPCRA. *AtPMEPCRA* may reinforce the pectin structure in order to mechanically counteract the effects of spaceflight microgravity. The altered growth parameters observed in mutants following spaceflight is probably because of altered pectin stiffening. Here, we also proposed that the defective pectin modifications caused by *atpmepcra* mutants could be a trigger to activate the ABA-related signaling pathway in Arabidopsis. The upregulated expression of the ABA biosynthesis genes *NCED2* and *NCED3* in the F_1_ offspring of microgravity-grown *atpmepcra* mutants supports this idea (Figure 8E). Treatment with pectinase has been shown to induce JA production, suggesting that enzymatic pectin breakdown activated JA biosynthesis (Engelsdorf et al., 2018). The results also support the role emerging of the ABA signaling pathway in cell wall remodeling in response to the spaceflight microgravity environment.

Changes in genomic DNA methylation patterns are usually closely related to transgenerational effects in genetically uniform population of plants. This led us, in our previous study, to systematically compare the levels of 5-methylcytosine (5-mC) in genomic DNA isolated from the parent generation and the progeny of ABA-treated and -untreated wild-type plants (Xu et al., 2021; Xu et al., 2018). In addition, the frequency of various growth phenotypes and ABA response of the offspring plants, cultured under non-ABA conditions, was checked. Interestingly, in the current study, the *atpmepcra* gene mutations allowed recovery of the observed phenotype in the post-spaceflight F_1_ generation compared with the control F_1_ population derived from parent plants grown on Earth. This finding prompted us to consider whether the *AtPMEPCRA* gene may also be affected epigenetically by DNA methylation. We checked the DNA methylation patterns in the *AtPMEPCRA* gene locus of the F_0_, F_1_, and F_2_ generations. The *AtPMEPCRA* gene locus was less methylated in gene body (and expressed to a lower level) in the F_1_ generation from the seedlings that underwent spaceflight than in the generation from seedlings that stayed on Earth. Disabling *AtPMEPCRA* by mutation also appeared to impact the adaptation of the wild-type plants to spaceflight. We conclude that space microgravity leads to transgenerational altered genomic flexibility that may increase the potential for adaptation to the specific requirements of spaceflight.

Pectin, one of the main components of plant cell wall, is de-esterified by the pectin methylesterase (PME). PME activity plays a key role in plant development and stress response. The ability of AtPMEPCRA functioning effectively at higher, more alkaline pH levels may indicate that a molecular adaptation has evolved that is conducive to pectin-mediated cell wall strengthening, which is valuable in response to spaceflight microgravity environments. Acidification of the apoplast is known to activate the cell wall-loosening enzymes, which cause the cell to expand (Barbez et al., 2017). Another hypothesis to be addressed is that AtPMEPCRA may play a role in maintaining cell wall integrity. The proteins pathogen recognition receptors (PRRs), wall-associated kinase 1 (WAK1), and the receptor-like kinase FERONIA (FER) all prioritize binding to de-methylesterified cross-linked pectin (Decreux and Messiaen, 2005; Feng et al., 2018). *AtPMEPCRA* promotes the binding of certain PRRs to pectin by producing blockwise de-methylesterified pectin. The data demonstrate the impact of direction and intensity changes of gravity on cell wall metabolism during plant gravitropism and on cells in the state of weightlessness. It is assumed that the maintenance of cell shape requires a balance between cell wall rigidity and cell turgor. Therefore, changes in gravity may affect cell growth by influencing the balance. As a result, the supporting tissue system of plants is weakened in the process of adapting to the microgravity environment, which may further affect the plant cell expansion, flowering time, root growth, and development. In summary, the results of these investigations are beneficial for understanding the mechanism of plant adaptation to microgravity.

### Limitations of the study

As with any research of spaceflight, there are many limitations. For example, true replicates are limited, as this would require repeat flights, which is often not possible; the seedlings were only exposed to 11 days, thus not truly grown entirely in spaceflight. Also, whether the effects are due to spaceflight or other environmental perturbations the seedlings experienced particularly on takeoff and landing or on the satellite is impossible to resolve without gravity-treated control samples grown in space. However, despite these limitations, I believe these studies are a step toward understanding the mechanisms plants respond to unique environments.

## Data availability

The data that support the findings of this study are available from the corresponding author upon reasonable request.

## STAR★Methods

### Key resources table

**Table:** 

REAGENT or RESOURCE	SOURCE	IDENTIFIER
**Bacterial and virus strains**		

Agrobacterium tumefaciens GV3101	Xu et al. (2018)	N/A
Rosetta (DE3) chemically competent cells	Xu et al. (2018)	CAT#J43270

**Chemicals, peptides, and recombinant proteins**		

Citrus pectin	Sigma	CAT#9046-40-6
Methyl Red	Sigma	CAT #493-52-7
NaCl	BBI	CAT# 7647-14-5
Pectin from apple	Sigma	CAT#9000-69-5
ABA	Sigma	CAT#14375-45-2

**Critical commercial assays**		

Qiagen EpiTect Bisulfite Kit	Qiagen	CAT#591040
Plant Genomic DNA Purification Kit	GeneMark	CAT#DP022-150
HisTrap affinity chromatography column	GE Healthcare	CAT#17524701
ReverTra Ace M-MLV reverse transcriptase kit	Toyobo	CAT#TRT-101

**Experimental models: Organisms/strains**		

Arabidopsis: *atpmepcra-87*	NASC	Salk_121787
Arabidopsis: *atpmepcra-47*	NASC	Salk_067447
Arabidopsis: Col-0	N/A	N/A
Arabidopsis: *nced3*	Xu et al. (2020)	Transgenic Col-0
Arabidopsis: proAtPMEPCRA:GUS	This study	Transgenic Col-0
Arabidopsis: *nced2nced3*	Xu et al. (2020)	Transgenic Col-0
Arabidopsis: *abi4*	Lionetti et al. (2017)	Transgenic Col-0
Arabidopsis: *coi1*	Lionetti et al. (2017)	Transgenic Col-0
Arabidopsis: *sid2*	Lionetti et al. (2017)	Transgenic Col-0
Arabidopsis: *ein2*	Lionetti et al. (2017)	Transgenic Col-0

**Oligonucleotides**		

Provided in Tables S1–S3		

**Recombinant DNA**		

pro1300GN::GUS	This study	N/A
His-AtPMEPCRA	This study	N/A

**Software and algorithms**		

Prism 7 software	GraphPad	https://www.graphpad.com
ImageJ software	Fiji programme	https://imagej.net

### Resource availability

#### Lead contact

Further information and requests for resources and reagents should be directed to and will be fulfilled by the lead contact, Weiming Cai (wmcai@cemps.ac.cn).

#### Materials availability

This study did not generate new unique reagents.

### Experimental model and subject details

#### A. thaliana

Wild-type (Col-0) Arabidopsis and *pmepcra-87* (Salk_121787) and *pmepcra-47* (Salk_067447) mutant lines on Earth or in space germinated and grew for 11 d on 1/2 MS medium containing 3% sucrose and 0.9% agar, with a photoperiod of 16 h, under 120 μmol m^-2^ s^-1^ at 21°C ± 2°C. The two T-DNA insertion lines of *AtPMEPCRA* were ordered from The Arabidopsis Information Resource (TAIR). The spaceflight experiment was part of the SJ-10 spaceflight project which was launched from the Jiuquan satellite launch center in April 2016. The sample preparation before launch was similar to the launch conditions described in the previous paper (Xu et al., 2018). The specifications of the equipment box, plant growth parameters and SJ-10 satellite flight parameters were as described in the previous papers (Xu et al., 2021).

#### Bacterial strains

Agrobacterium tumefaciens GV3101 and Rosetta (DE3) chemically competent cellswas cultivated in LB medium supplemented with appropriate antibiotic in a shaker at 28°C.

### Method details

#### 3D-clinostat experiment

A 3D clinostat (SM-31 tow-axis driving clinostat) was designed and constructed for the simulated weightlessness (made by Center for Space Science and Applied Research, Chinese Academy of Sciences, 2005). The main power of rotation was provided by two geared stepping motors and the sample stage was three dimensionally rotated by changing the rate and direction of rotation at random from 1 to −1 (reverse direction) rpm every 1 min. The module was illuminated by light banks made up of fluorescent lamps with a photoperiod and temperature conditions as the SJ-8 space experiment described above (Zheng et al., 2008).

#### Total RNA isolation and real-time quantitative PCR (qPCR)

Total RNA was extracted from 11-d-old seedlings of *A. thaliana* grown on 1/2 MS solid medium plates, using RNeasy Plant Mini Kit (Qiagen, Hilden, Germany), and the quality and quantity of RNA samples were evaluated using a 2100 Bioanalyzer (Agilent Technology Co., Ltd. USA). cDNA was synthesized using oligo (dT) 18 primer (ThermoFisher Scientific, USA) and ReverTra Ace M-MLV reverse transcriptase kit (Toyobo, Japan), according to the manufacturer's recommendation. The gene primers for qPCR are listed in Table S1.

#### DNA extraction and PCR program for bisulfite sequencing for methylation studies

The total DNA was extracted using the method described previously (Xu et al., 2018). Three biological replicates per sample were used for genome bisulfite sequencing analysis from spaceflight and Earth samples. Samples were pretreated with the Qiagen EpiTect Bisulfite Kit (Qiagen, Hilden, Germany), and the Plant Genomic DNA Purification Kit (GeneMark, Taiwan) was used to extract genomic DNA. The PCR program settings for cloning fragments were as described previously (Xu et al., 2018).

#### Construction of proAtPMEPCRA::GUS Vector and Staining

The 2,500-bp region upstream of ATG (pro*AtPMEPCRA*), representing the promoter region, was amplified from the Arabidopsis genome and cloned into the pro1300GN::GUS vector. The wild-type Arabidopsis plants (Col-0) were transformed. GUS staining was used to investigate the expression of AtPMEPCRA in T2 generation transgenic plants. After incubating in GUS staining solution overnight at 37°C, samples were decolorized in 75% ethanol and observed under a light microscope.

#### Determination of PME activity

PME activity was assayed according to the method of Solecka (Solecka et al., 2008). The reaction mixture contained 0.5% (w/v) citrus pectin (Sigma, Germany), 0.2 M NaCl and 0.15% (w/v) Methyl Red (Sigma, Germany), pH 6.8. Protein sample (50 μL) was added to 950 μL of reaction mixture in a micro cuvette. Protein sample was determined by the Bradford protein assay method (Bradford, 1976). The change in color from yellow to red after incubating at 25°C for 20 min was measured spectrophotometrically at 525 nm. A calibration curve was obtained by adding 1–6 x 10^-3^ mM HCl to the reaction mixture. Recombinant AtPMEI1 was expressed in *P. pastoris* (Raiola et al., 2004). *B. cinerea* PME activity was induced in the *B. cinerea* culture by adding 0.5% apple pectin (wt / vol; 76,282; Sigma-Aldrich) and after 12 hr the culture filtrate was collected (Lionetti, 2015).

#### His-AtPMEPCRA protein expression in *E. coli*

The *AtPMEPCRA* sequence was amplified and cloned into pET-30a (+) vector. The recombinant vector was used to transform *E. coli* BL21-CodonPlus (DE3) cells. Overnight cultures of transformants were grown in LB medium at 37°C to reach OD_600_ values of 0.6–0.8, and 0.1 mM isopropyl β-D-1-thiogalactopyranoside was added and incubated at 28°C to induce expression for 4 h. His-AtPMEPCRA was purified by using HisTrap affinity chromatography column (GE Healthcare, USA) according to the manufacturer’s instructions.

#### Flowering time analysis

The flowering time was measured as the time from germination to the opening of the first flower bud of each Arabidopsis plant; the number of rosette leaves at flowering was also counted. The plants were grew under a photoperiod of 16 h light and 8 h dark, under 120 μmol m^-2^ s^-1^ at 21°C ± 2°C.

#### Root gravitropism assay

Four-day-old seedlings grown on solid ½ MS medium in a Petri dish were cultured horizontally for a period of time, and the root bending angle on plate was calculated as described previously using Image J software (https://imagej.net).

#### ABA treatment

For seed germination and cotyledon greening rate assays (visually), Col-0 and *atpmepcra* mutant seeds were treated with 0.0, 0.5 or 2 μM ABA on half-strength MS solid medium for 7 d. All Col-0 and *pmepcra* mutant seeds used were the next generation of seedlings (F_1_ seedlings) which grew in space or on Earth.

### Quantification and statistical analysis

The quantitative data are expressed as mean ± SE or mean SD of at least 3 (indicated in the figure legends, when the number was more than 3) independent experiments. Statistical analysis was performed using student’s *t* test. Values of p < 0.05 were considered statistically significant.

## Data Availability

All data reported in this paper will be shared by the lead contact upon request. This paper does not report original code. Any additional information required to reanalyze the data reported in this paper is available from the lead contact upon request.
