# Supplementary material for: Pectin methylesterase gene AtPMEPCRA contributes to physiological adaptation to simulated and spaceflight microgravity in Arabidopsis
Source: iScience. 2022 Apr 29;25(5):104331. doi: 10.1016/j.isci.2022.104331 (PMC9118689; doi:10.1016/j.isci.2022.104331)
Supplement: Document S1. Figures S1–S4 and Tables S1–S4 [file mmc1.pdf]

iScience, Volume *25*

Supplemental information

Pectin methylesterase gene *AtPMEPCRA* contributes to physiological adaptation to simulated and spaceflight microgravity in Arabidopsis

Peipei Xu, Haiying Chen, Jinbo Hu, Xiaocheng Pang, Jing Jin, and Weiming Cai

Supplementary information

Article title: Pectin methylesterase gene *AtPMEPCRA* contributes to physiological adaptation to simulated and spaceflight microgravity in Arabidopsis

Peipei Xu[1#], Haiying Chen[1#], Jingbo Hu[1#, 2], Xiaocheng Pang[1], Jing Jin[1] & Weiming Cai[1*]

[1]Laboratory of Photosynthesis and Environment, CAS Center for Excellence in Molecular Plant Sciences, Shanghai Institute of Plant Physiology and Ecology , Chinese Academy of Sciences, No. 300 Fenglin Road, Shanghai 200032, China

[2]University of Chinese Academy of Sciences, Beijing 100039, China

[*]Corresponding author.

[#]contributed equally.

Telephone.86-21-54924248

Fax.86-21-54924015

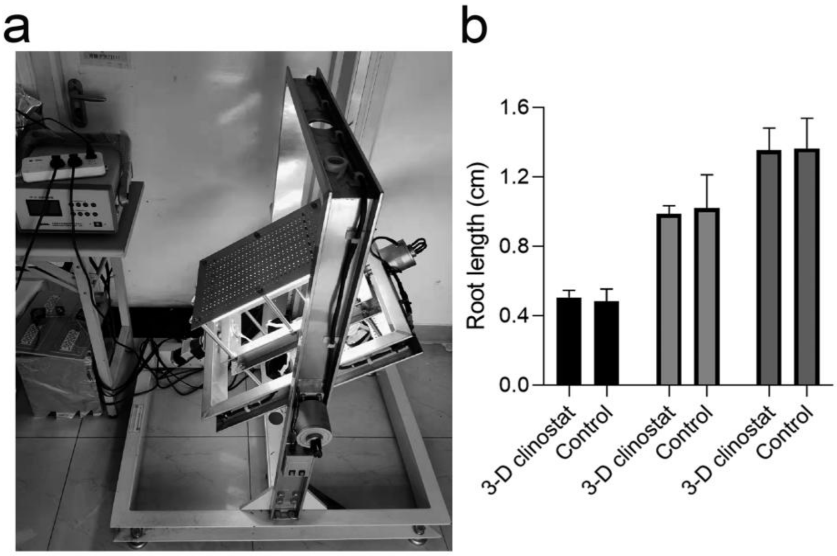

Figure S1. Wild-type Col-0 plants were exposed to simulated microgravity in a 3-D clinostat (related to Figure 1).

a. The three-dimensional (3-D) clinostat equipment we used. **b**. Wild-type Col-0 plants were exposed to simulated microgravity in a 3-D clinostat. Seedlings were harvested after 4-8 days of simulated microgravity treatment. Root length was not significantly different from that of gravity-treated control plants. The bar represents SD. The main power of rotation was provided by two geared stepping motors and the sample stage was three dimensionally rotated by changing the rate and direction of rotation at random from 1 to −1 (reverse direction) rpm every 1 min. The module was illuminated by light banks made up of fluorescent lamps with a photoperiod and temperature conditions as the SJ-8 space experiment described (Zheng et al., 2008).

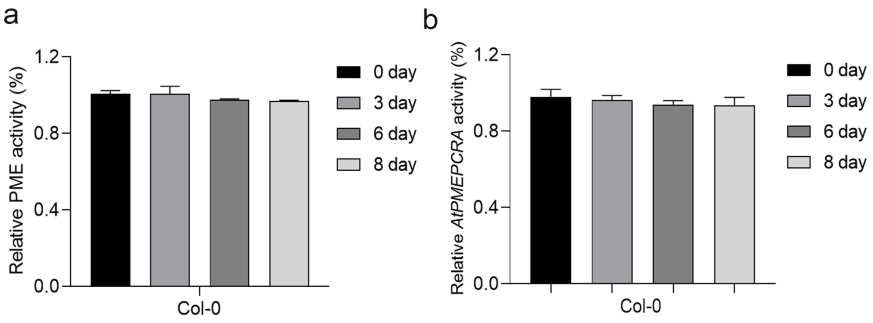

Figure S2. Wild-type Col-0 plants were exposed to horizontal rotation provided by a single axis of the clinostaty (related to Figure 1).

a. We have supplemented the horizontal rotation experiment provided by a single axis of the clinostat. Seedlings were harvested after 3, 6 and 8 days of horizontal rotation treatment. PME activity was not obviously inhibited under the challenge of horizontal rotation treatment. 0 day as a 100% control for the respective group. Bars indicate SE. **b**. qPCR analysis of gene expression pattern of *PMEPCRA* in Arabidopsis after exposure to horizontal rotation treatment. Bars indicates SE from three independent experiments.

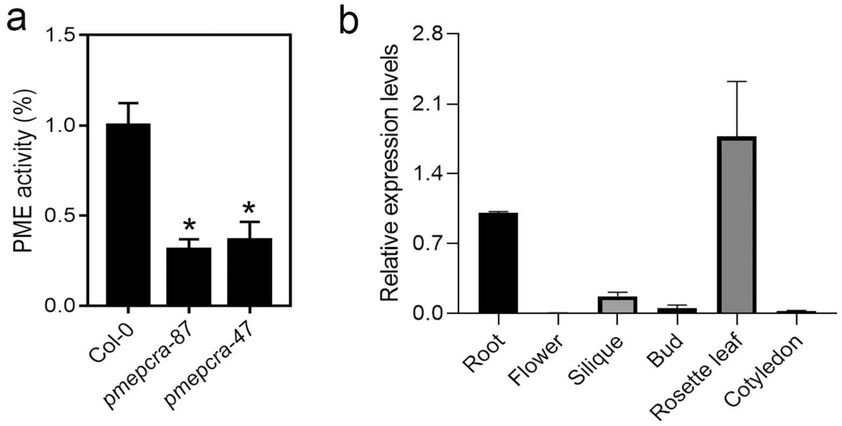

Figure S3. Analysis of PME activity and relative expression level of *AtPMEPCRA* gene in various tissuesy (related to Figure 1 & Figure 3).

a. Relative activity of PME activity in Col-0 and *atpmepcra* mutants background. The total protein was extracted and the activity of PME was determined. **b**. Identification of relative AtPMEPCRA gene expression level in various tissues of plant. The bar represents SD. *indicates statistically significant difference using Student's *t* test (p<0.05).

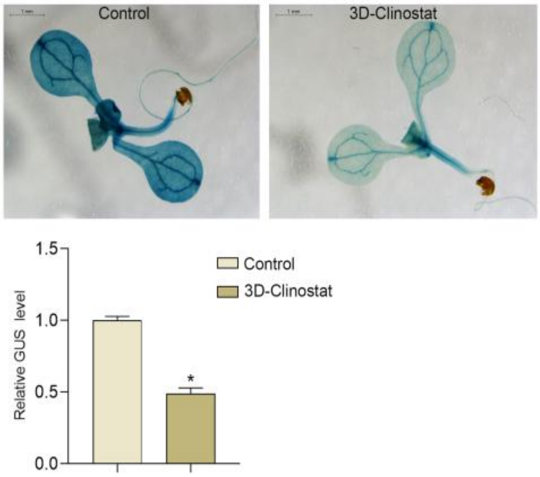

Figure S4: **pPMEPCRA::GUS expression analysis in the control and after simulated microgravity treatmenty (related to Figure 3).**

The pPMEPCRA::GUS expression pattern did not change after simulated microgravity treatment, but the GUS level was significantly suppressed, which was consistent with the results of quantitative RT-PCR analysis.

Table S1. Gene-specific primers used in the mutant screeny (related to Figure 3).

**Genes**	**Primers (Sequence 5'-3')**
SALK_121787 LP:	TGTCGTCGTTAGTGTGTCGAG
SALK_121787 RP:	AACACGTGCAATAGGTCAAGG
SALK_067447 LP:	AGGAGCAAGATCCGAACTAGC
SALK_067447 RP:	TGATGAGTTCGATGGAGGAAC

Table S2. Gene-specific primers used in the RT-qPCR analysisy (related to Figure 1).

**Genes**	**Primers (Sequence 5'-3')**
AtACT2 qRT F:	5'-TCAGATGCCCAGAAGTCTTGTT-3'
AtACT2 qRT R:	5'-CCGTACAGATCCTTCCTGATATC-3'
AtTUB2 qRT F:	5'-GAGCCTTACAACGCTACTCTGTC-3'
AtTUB2 qRT R:	5'-ACACCAGACATAGTAGCAGAAA-3'
AtPMEPCRA qRT F:	5'-CGTATCAAGACACGCTCTACAC-3'
AtPMEPCRA qRT R:	5'-CGATGTCGCAGTTCTGGAATA-3'
AtNCED2 qRT F:	5'-TCATACTCAGCCGCCATTATC-3'
AtNCED2 qRT R:	5'-GTGTGAAGCGCAGATGAAAC-3'
AtFT qRT F:	5'-TGGAGAAGACCTCAGGAACT-3'
AtFT qRTR:	5'-CAGTCACCAACCAATGGAGA-3'
AtFLC qRT F:	5'-GCCAAGAAGACCGAACTCAT-3'
AtFLC qRT R:	5'-TCTGCTCCCACATGATGATTATT-3'
AtABI4 qRT F:	5'-AATGGTGGGACCTCTATGTTATG-3'
AtABI4 qRT R:	5'-CCCATCTGGACCATCTGATTT-3'
AtNCED3 qRT F:	5'-GCATAATCCTCTCCGGCTAAA-3'
AtNCED3 qRT R:	5'-TGGAGAATCTTGCTCGTGTG-3'

Table S3. The primers used in BSP analysisy (related to Figure 6).

**Transgenes**	**Primers (Sequence 5’-3’)**
PMEPCRA BSP 1F:	AAGATTTATGTTAAGTTTTTTTGTT
PMEPCRA BSP 1R:	ATATAACTCAAAATTATAATTTCCTCC
PMEPCRA BSP 2F:	GGTAAAGTATTAGGTTGTGGTTTTA
PMEPCRA BSP 2R:	ACATATTCTTTTACCCAACTCCAAA

Table S4. The alteration of DNA methylation in PME family under microgravity conditiony (related to STAR Methods).

AGI number	UniProktKB	PME domain	PME class	PME type	Annotation	Diff Methy F0 (S0-G0)	Diff Methy F1 (S1-G1)	Expression Changes (F1)
At2g19150	*AtPME10*	p	A	II	intron 2 of 4	-0.225	-0.002	No significant
At2g36700	*AtPME14*	p	A	II	exon 3 of 5	0.280	0.003	No significant
At1g11590	*AtPME19*	p	C	I	TTS	-0.323	-0.011	No significant
At3g10710	*AtPME24*	p	C	I	intron 10 of 12	-0.274	-0.004	No significant
					exon 11 of 13	-0.312	-0.002	No significant
At5g27870	*AtPME28*	p	B	I	TTS	-0.331	-0.005	No significant
At3g27980	*AtPME30*	p	C	I	TTS	-0.328	-0.004	No significant
At3g43270	*AtPME32*	p	D	I	promoter-TTS	-0.284	-0.008	No significant
At4g03930	*AtPME42*	p	C	I	promoter-TTS	-0.194	-0.007	No significant
At5g04960	*AtPME46*	p	C	I	Promoter-TTS	0.521	0.011	No significant
At5g07410	*AtPME48*	p	A	II	TTS	0.246	0.004	No significant
At5g07420	*AtPME49*	p	A	II	promoter-TTS	0.206	0.004	No significant
At5g09760	*AtPME51*	p	A	I	TTS	-0.345	-0.006	No significant
At5g55590	*AtPME62*	p	A	II	TTS	-0.360	-0.007	No significant
